# How Does the Implicit Awareness of Consumers Influence the Effectiveness of Public Service Announcements? A Functional Near-Infrared Spectroscopy Study

**DOI:** 10.3389/fpsyg.2022.825768

**Published:** 2022-03-14

**Authors:** Jialin Fu, Xihang Li, Xi Zhao, Keyi Zhang, Nan Cui

**Affiliations:** ^1^College of Economics and Management, Zhengzhou University of Light Industry, Zhengzhou, China; ^2^Economics and Management School, Wuhan University, Wuhan, China

**Keywords:** neuromarketing, functional near-infrared spectroscopy (fNIRS), PSAs, dorsolateral prefrontal cortex (dlPFC), implicit awareness, purchase decision

## Abstract

A large number of scholars have conducted detailed studies on the effectiveness of commercial advertising by using neuroimaging methods, but only a few scholars have used this method to study the effectiveness of public service announcements (PSAs). To research the relationship between the effectiveness of PSAs and the audience’s implicit awareness, functional near-infrared spectroscopy (fNIRS) was employed to record the neural activity data of participants in this study. The results showed that there was a correlation between activation of dorsolateral prefrontal cortex (dlPFC) and the effectiveness of PSAs; The activation of the dlPFC could also be used as an indicator to represent the appeal of advertising content. The results means that neuroimaging tool can also be used to investigate the effectiveness of PSAs, not just commercial advertisements and a few PSAs study, and that neural activity can predict and improve the effectiveness of PSAs before they are released.

## Introduction

Advertising effectiveness is one of the most important indicators for advertisers. For all advertising campaigns, consumers are the recipients of advertising, and the effectiveness of advertising is ultimately reflected by the behavior of consumers (Ramsoy, 2019). To obtain better advertising outcomes, enterprises design different advertisements to promote new products and hire celebrities to endorse their products at great cost (Clark et al., 2018). Some companies have established a good brand image and gained good revenue through these methods, but there are also some companies that have not met their expectations (Huang et al., 2018). In addition to commercial advertisements, public service announcements (PSAs) focus on advertising effectiveness.

The existing studies of the effectiveness of PSAs are mostly focused on acquired immunodeficiency syndrome (AIDS) prevention (David and Cindy, 1996; Wang and Arpan, 2008), smoking cessation (Shen, 2010; Wang et al., 2013), drug rehabilitation (Fishbein et al., 2002), traffic safety (Santa and Cochran, 2008), etc. All of these studies evaluate the effectiveness of PSAs by understanding people’s views on PSAs through interviews, questionnaires, and other forms of self-report. However, self-repots sometimes do not reflect what people really think (Dmochowski et al., 2014). Evaluating the effectiveness of PSAs solely on self-reports may lead to wrong conclusion, which will greatly reduce the effectiveness of them.

The emergence of neuromarketing offers a new approach to the study of advertising effectiveness. Telpaz et al. (2015) believe that people are often reluctant to express themselves or unable to express themselves correctly, but their neural activity, heart rate, and other implicit awareness indicate what they are truly thinking. Neuromarketing, unlike traditional marketing approaches, employs neuroimaging tools to record consumers’ neural responses to products, brands, and advertisements and can be used to analyze neural data to explain and predict consumers’ decisions (Lim, 2018; Hakim and Levy, 2019). Several scholars have proved that neural activity can provide higher prediction accuracy than self-report (Dmochowski et al., 2014; Boksem and Smidts, 2015; Telpaz et al., 2015; Barnett and Cerf, 2017; Chan et al., 2019).

Some researchers have introduced neuroimaging tools into the study of PSAs. They have studied the predictability of advertising effect in PSAs (Falk et al., 2012), the relationship among advertising effect, content, and neural activity (Wang et al., 2013), and the indicators that distinguish effective PSAs from ineffective PSAs by neural activity (Cartocci et al., 2018). However, despite the abundance of previous studies, three problems remain. First, most of the previous studies were posterior and did not reveal indicators that could improve effectiveness of PSAs; Second, almost all of previous studies using neuroimaging tools were on anti-smoking PSAs, and the applicability of the findings to other types of PSAs remains to be tested; Third, functional MRI (fMRI), the neuroimaging machine used in the previous studies, is extremely expensive and large, making it difficult to scale up widely.

Therefore, in this study, functional near-infrared spectroscopy (fNIRS), a new, cheaper and more portable neuroimaging tool, was used to measure participants’ neural activity while viewing PSAs, with the goal of finding indicators that could improve the effectiveness of PSAs. An experiment on agricultural PSAs was conducted to find indicators of neural activity that would improve the effectiveness of PSAs. In this paper, the progress of related studies is summarized and hypotheses are presented in the Literature Review. Then the experimental design and data processing are described in the Method. The results of data analysis are shown in the Results. Then after that, the findings are discussed and compared with literature in the Discussion. Finally, in the Conclusion, conclusions are stated and possible topic selections for future research are suggested.

## Literature Review

Advertising effect has received much attention in neuromarketing, and numerous studies have investigated the relationship between neural activity and advertising effect. Previous studies have shown that neural activity is a better predictor of advertising effect than self-report. By analyzing neural activity of the ventral striatum, Berns and Moore (2012) found that the activation within the ventral striatum was correlated with the sales of music albums, while self-report was not. Not limited to music albums, the advertising effect of printer poster can also be accurately predicted. In analyzing the neural activity of participants’ viewing advertising poster for chocolate bars at different times, Kühn et al. (2016) assigned different weight to different brain region and successfully predicted the sale ranking of chocolate bars at different times, which was not possible for self-report.

Within the field of PSAs, several studies have focused on investigating the relationship between anti-smoking PSAs effectiveness and neural activity. One study found that neural activity was also a much better predictor of PSAs than self-report. Falk et al. (2012) found that medial prefrontal cortical neural activity, while participants viewed anti-smoking PSAs was associated with the number of calls to an anti-smoking hotline. Previous studies have found that the content and format influence the effectiveness of PSAs (Dillard and Peck, 2000; Santa and Cochran, 2008). Several scholars have studied how content and format affect the effectiveness of PSAs through neuroimaging tools. By comparing participants’ neural activity and behavior after viewing anti-smoking PSAs, Wang et al. (2013) found that neural activity evoked by PSAs with different content differed significantly in the inferior frontal gyrus, the precuneus and the dorsomedial prefrontal cortex (dmPFC), and that neural activity in dmPFC could predict the urine cotinine levels 1 month later, which reflected participants’ smoking intensity. Through measurement and analysis of multiple instruments, Cartocci et al. (2018) found that effective ads focused on visual elements while ineffective ads focused on text.

Interestingly, the prefrontal cortex (PFC) has been frequently mentioned in studies of PSAs effect. Functionally, the PFC can be divided into three parts, the orbitofrontal cortex (OFC), ventral prefrontal cortex (vPFC), and the dorsal prefrontal cortex (dPFC; Parnamets et al., 2020). The OFC is the brain region associated with value assessment, the vPFC is the brain region associated with emotions, and the dPFC is the brain region associated with working memory and rational thinking (Plassmann et al., 2015; Karmarkar and Plassmann, 2019). Notably, the PFC is also the main area of interest for scholars who have used fNIRS for advertising effect. Most of the existing studies of advertising effects *via* fNIRS chose the dorsolateral prefrontal cortex (dlPFC) as the observed brain region. Some scholars have verified the reliability of fNIRS in advertising effect research by repeating previous fMRI experiments (Krampe et al., 2018; Gier et al., 2020; Meyerding and Mehlhose, 2020). Gier et al. (2020) repeated Kühn et al. (2016) study on the advertising effect of chocolate bars by measuring the neural activity of dlPFC, and obtained a high accuracy. Some scholars have also used fNIRS to measure neural activity in the dlPFC to reveal a variety of factors that influence the effectiveness of advertising, such as gender differences (Duan et al., 2021), preference differences (Qing et al., 2021), etc. However, fNIRS also has disadvantages. Limited by the penetrating ability of NIR light, fNIRS cannot measure neural activity in deep brain regions, such as the ventral medial prefrontal cortex.

Therefore, in this study, the dlPFC was selected as the target observation region to collect neural activity of participants while viewing PSAs. The following hypothesis has been proposed that the dlPFC activity can predict the effectiveness of PSAs and help improve the advertising effect.

## Materials and Methods

### Participants

Fourteen males and 16 females participated in this experiment (age *M* = 24.47, years, *SD* = 1.69). All participants attended Zhengzhou Light Industry University, were right-handed, had normal or corrected-to-normal vision, no brain injury, no history of psychiatric disorders, no recent use of tranquilizers, and no previous participation in neuroscience experiments. The experiment was approved by the ethics committee of the university.

### Materials

The year 2021 is the first year that China has declared that everyone is free from poverty, and almost all Chinese people have focused on the cause of poverty eradication. China Central Television (CCTV) has released many PSAs to help poor regions promote their special products and industries during the fight against poverty. Since all Chinese people are aware of the project of fighting poverty, we chose PSAs related to this project as experimental materials. The PSAs released by CCTV contain three themes: tourism, represented by landscapes, agricultural products, represented by fruits, vegetables and meat, and traditional handicrafts. Considering that the participants were university students, fruit and milk ads were chosen as the stimulus material for this experiment in order to be more relevant to reality. Ten different ads were selected from the agricultural PSAs released by CCTV from January 2020 to December 2020^1^, as shown in Table 1.

**Table 1:** Task materials.

Number	Agricultural products	Origin
v1	Carambola	Fujian
v2	Red date	Xinjiang
v3	Red kiwi	Jiangxi
v4	Apple	Sichuan
v5	Navel orange	Chongqing
v6	Pomelo	Fujian
v7	Passion fruit	Fujian
v8	Milk	Gansu
v9	Pitaya	Guangxi
v10	Roxburgh rose	Guizhou

### Task Design

In this experiment, participants were told that purchasing the products in PSAs was considered as a willingness to support the anti-poverty project, and participants’ willingness to purchase the products in the PSAs was considered as an indicator of advertising effect. To determine what factors can enhance advertising effect, a control group containing price was added into the experiment, which was designed whether people would actually behave as expected in PSAs when they are in their lives. After the experiment, participants in the experiment group and control group were randomly invited to participate in a telephone interview, with the aim of finding the reason for the difference in the result between two groups.

The group that did not include price was named group A and the group that included price was named group B. Before the experiment began, participants were randomly assigned to group A and group B. Participants in group A were told that they did not need to take price into account, and they could make decisions based on their own preferences. In contrast, participants in group B were told that the product price would be displayed during the decision phase and that they should make a decision choice based on their real needs.

**Figure 1:**
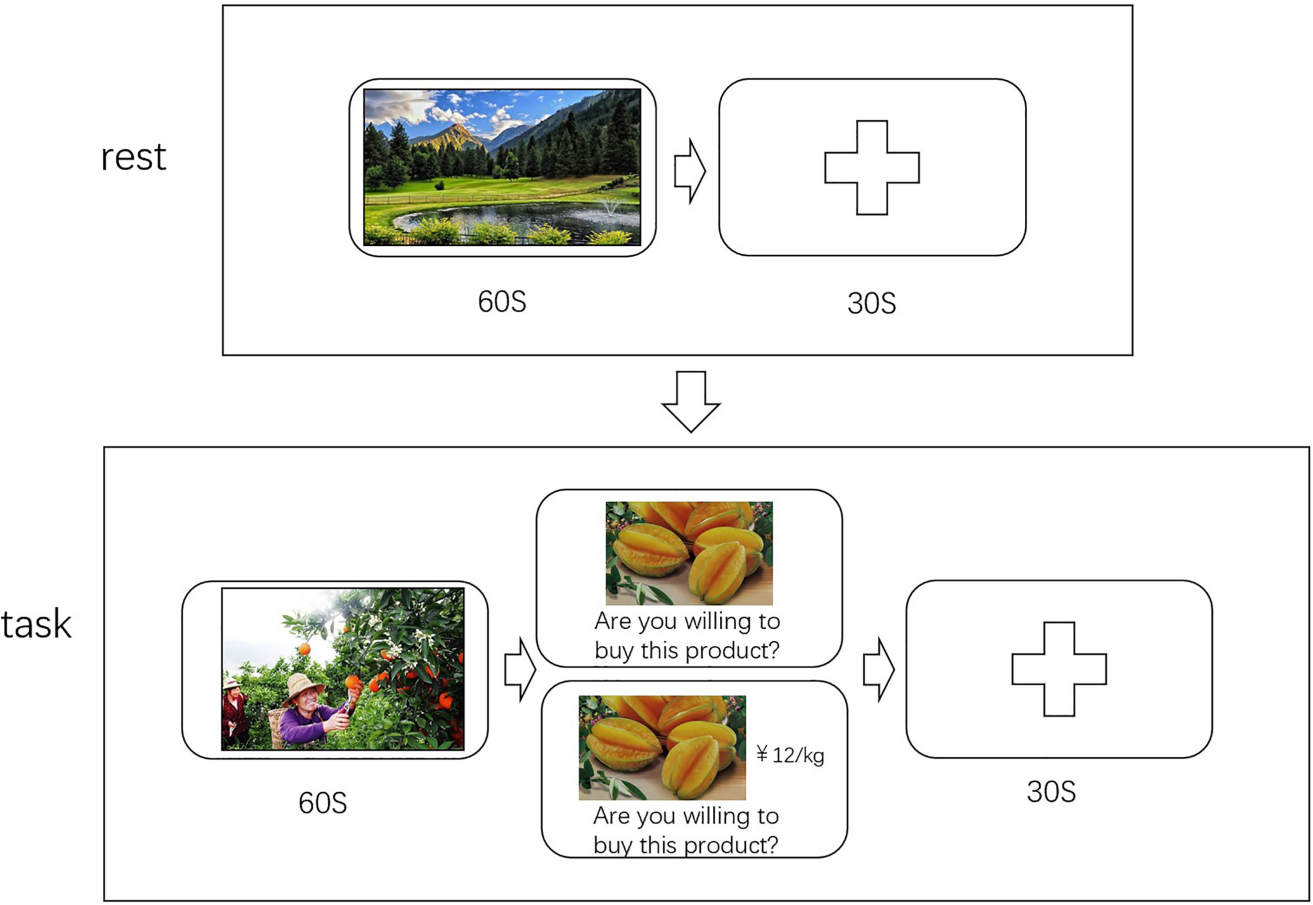
The top half of the picture “rest” is the first part of the experiment, where participants view the picture for 60 s and then rest for 30 s before moving on to the second part. The bottom half of the picture, “task,” shows the process of each trial in the second part, including an ad and a decision-making session.

The experiment consists of two parts, the first part collected neural data when participants were resting and the second part collected neural data when participants were watching the advertisement. In the first part, a landscape picture that lasted 60 s would appear on the screen, and participants simply watched the picture without any reaction. After the picture disappeared, the participants rested for 30 s and then entered the second part. The second part consisted of 10 trials, each of which containing an advertising video and a decision-making session. The ads lasted 60 s, and the decision-making session had no time limit until the participants made a choice. In decision-making session, only product pictures in the ads appeared in group A while product pictures and prices appeared in group B. There was a 30-s break at the end of the decision-making session, and the experimental flow is shown in Figure 1.

### Data Acquisition and Processing

**Figure 2:**
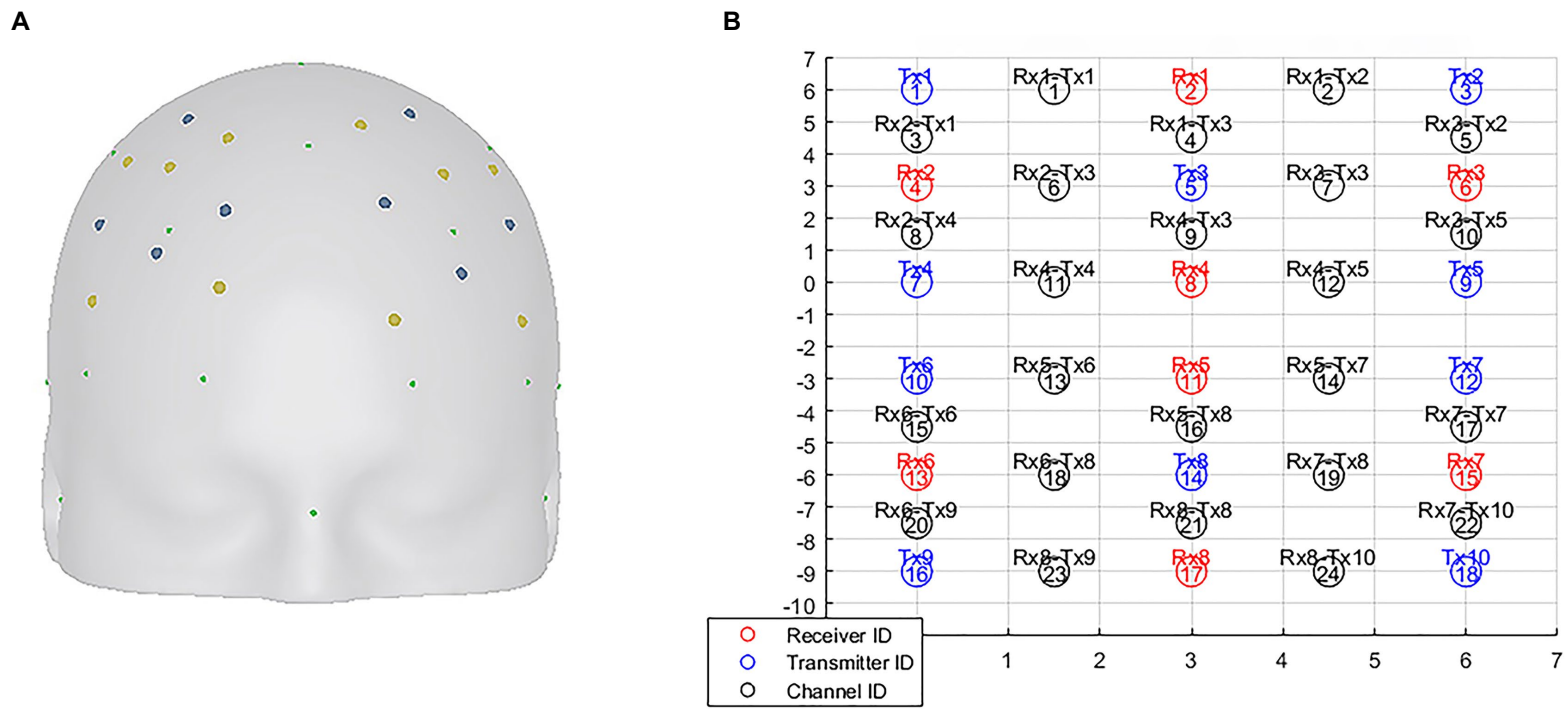
The locations of the transmitters and receivers are shown in **(A)**, with the yellow dot being the transmitter and the blue dot being the receiver. The distribution of each channel is shown in **(B)**, with channels 1–12 distributed in the right hemisphere and channels 13–24 in the left hemisphere. Channels 4, 6, 7, and 9 were selected as analysis channels for the right hemisphere, and channels 16, 18, 19, and 21 were selected as analysis channels for the left hemisphere.

The portable fNIRS used in the experiments was Artinis Brite 24 (10 transmitters and eight receivers), which emits NIR light at 762 and 841 nm and has a sampling rate of 10 Hz. Transmitter and receiver were separated from each other by a distance of 3 cm in order to guarantee signal quality. They were placed with reference to the 10–20 standard EEG positions, centered on F3 and F4 and symmetrically distributed along the central sulcus, as shown in Figure 2. E-Prime 3.0 was used to present the experimental stimuli, and Oxysoft (v3.2.72) was used as data collection software.

NIRS_KIT (Hou et al., 2021) was used to process the raw data and analysis. The pre-processing of the raw data went through the following steps: first detrending of the raw data, then Motion correction of the data by TDDR method, and finally band-pass filtering of the data by IIR method at 0.01–0.1 Hz. The preprocessed data were entered into the data analysis phase, and as oxyhemoglobin (HbO) correlates more with cerebral blood flow than deoxyhemoglobin (Hb; Strangman et al., 2002), only HbO was focused on in the next analysis.

For every participant, a general linear model (GLM) was set up to model neural activity during the experimental task. The model contained 11 parameters, respectively, “rest” and v1–v10. Next, for each participant’s neural data, v1–v10 was compared with “rest” to calculate the beta value, which was named condition 1–condition 10, respectively. Finally, in order to investigate the dlPFC activation induced by ads at the group level, one-sample *t*-test was performed for all participants’ beta values in condition 1–condition 10. Bonferroni correction was used to correct the *t*-test results. The ranking method of activation results refers to Kühn et al. (2016) and Gier et al. (2020). Channel 4, 6, 7, 9, 16, 18, 19, and 21 were selected as the comparison channels, and the highest value of corresponding *t*-value of the channels was selected as the ranking basis.

## Results

### Behavioral Results

**Figure 3:**
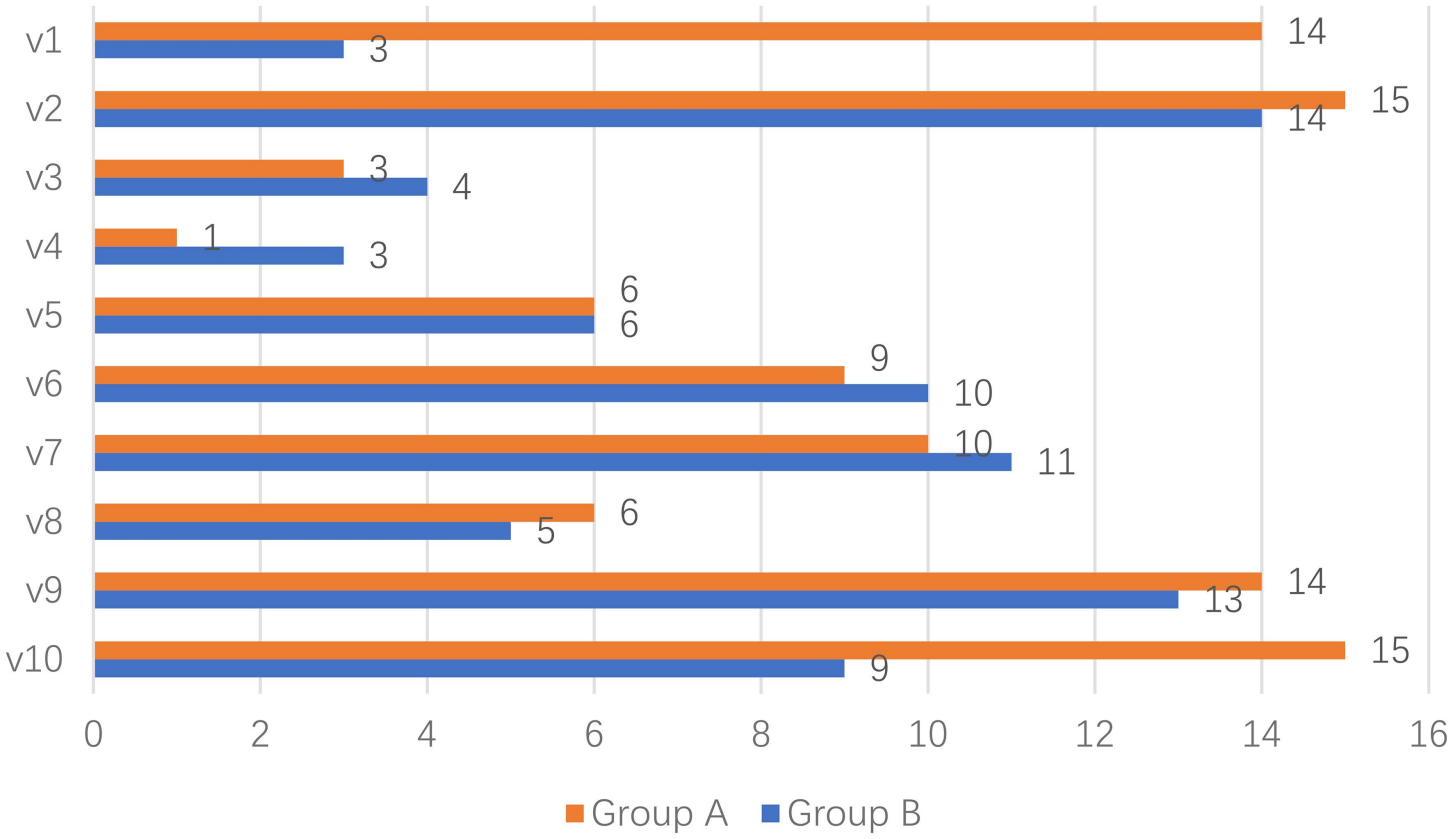
Purchase data for each group.

The decision-making session result data for each group was extracted from E-Prime 3 keylog (*M* = 9.30, *SD* = 5.17 for group A and *M* = 7.8, *SD* = 4.13 for group B), the results of which are shown in Figure 3. The various purchases of each product were ranked according to their purchase volume and ranked as follows: group A was v2, v10, v1, v9, v7, v6, v5, v8, v3, and v4; group B was v2, v9, v7, v6, v10, v5, v8, v3, v1, and v4. Independent samples *t*-test conducted with SPSS 20 was used to measure the difference between the groups. There was no significant difference in purchase volume between the two groups [t_(18)_ = 0.253, *p* > 0.05], but there was a large difference in purchase volume between the two groups for v1 and v10.

### fNIRS Results

**Figure 4:**
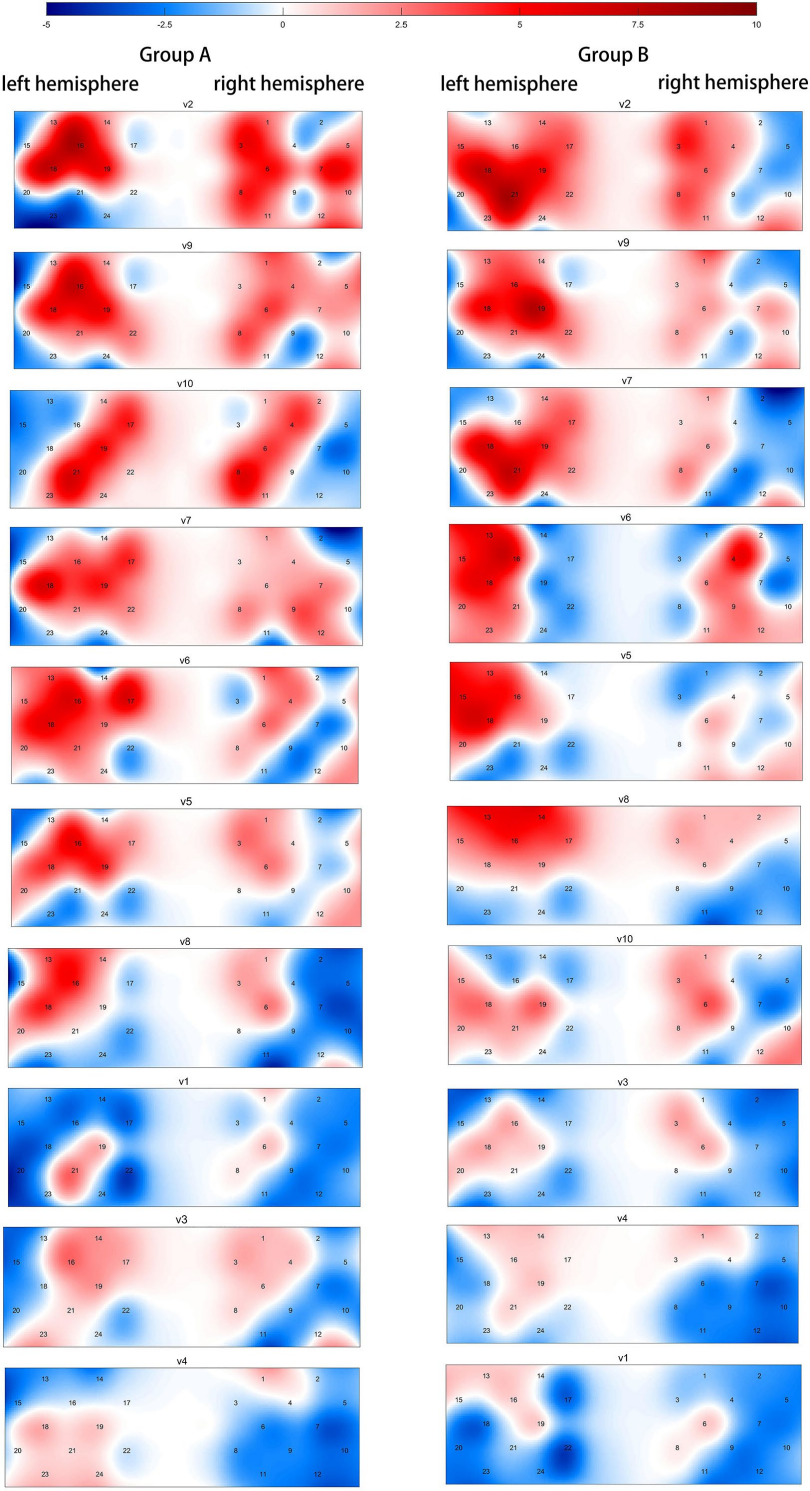
v1–v10 are ranked according to the peak *t*-value of the selected channel, with red representing the activation of dorsolateral prefrontal cortex (dlPFC) compared to the resting state. Compared to the resting state, v4 in group A and v3, v4, and v1 in group B were insignificant.

The peak *t*-values in the selected channels were used as the basis for ranking the degree of dlPFC activation, as shown in Table 2. The t-contrast activity map of each ad was sorted according to the peak value of the selected channel, as shown in Figure 4.

**Table 2:** The peak *t*-values of the selected channels.

Videos	Group A			Group B		
	T	*p*	Channel	T	*p*	Channel
v1	3.15	*p* < 0.01	21	1.09	*p* > 0.05	6
v2	7.67	*p* < 0.01	16	8.32	*p* < 0.01	21
v3	2.57	*p* < 0.05	16	1.73	*p* > 0.05	18
v4	1.63	*p* > 0.05	18	1.46	*p* > 0.05	19
v5	5.87	*p* < 0.01	16	6.39	*p* < 0.01	18
v6	6.33	*p* < 0.01	16	6.69	*p* < 0.01	16
v7	6.47	*p* < 0.01	18	7.27	*p* < 0.01	21
v8	5.54	*p* < 0.01	16	4.97	*p* < 0.01	16
v9	6.71	*p* < 0.01	16	8.32	*p* < 0.01	19
v10	6.62	*p* < 0.01	21	2.63	*p* < 0.05	6

It is worth noting that while most of the ads in the behavioral and fNIRS results are largely consistent in rankings, there are still some differences. v1 and v10 ranked 3rd and 2nd in the behavioral results of Group A, but 8th and 3rd in the fNIRS results. While v1 and v10 ranked 9th and 5th in the behavioral results of Group B, and 10th and 7th in the fNIRS results, the rankings of the two results are closer. To find the reason for this phenomenon, 10 participants were randomly selected to be interviewed.

### Interview Results

Compiling the interview transcripts revealed that almost all interviewees felt they were not familiar with the fruits introduced by v1 and v10. The origin of the carambola introduced in v1 and the roxburgh rose introduced in v10 are both very distant from the location of this experiment, and both fruits are very rare in the experimental location.

Interviewees felt they were more concerned about the taste of the fruits than the environment in which they were grown. In the ads, v10 introduced more about the taste of roxburgh rose, the way to consume and the deep processing products, while v1 focused more on the growing environment and the appearance of carambola. Moreover, because group A did not need to consider price when making decisions, almost all participants chose to try unfamiliar products; in contrast, group B needed to consider price when making decisions, so most participants made choices based on their preferences. Thus, the situation emerged that v1 and v10 differed in the ranking of behavioral and neural outcomes in group A, while they were more similar in group B.

## Discussion

The purpose of this study was to investigate whether consumers’ implicit awareness can predict the effectiveness of PSAs and help improve the effectiveness of PSAs. According to results, the neural activity collected by portable fNIRS accurately predicts the participants’ decision-making behavior after they viewed the PSAs. At the group level, the higher the participants’ dlPFC activation, the greater their purchase volume. This implies that activation of the dlPFC predicts the advertising effect of PSAs. This finding is consistent with the results of previous fMRI studies (Falk et al., 2012) and fNIRS studies (Gier et al., 2020). This means that the neuroimaging tool is not limited to anti-smoking PSAs, but can also be applied to other types of PSAs.

Activation of the dlPFC means that neural activity was significantly higher when participants viewed the PSAs compared to when they did not, implying that the PSAs had a significant effect on participants’ decision-making processes. The dlPFC is the brain area associated with working memory, value assessment, willingness to pay, and decision-making (Karmarkar and Plassmann, 2019). Previous research has suggested that the more attractive the stimulus is to the participant, the higher the activation of dlPFC in their decision-making process (Meyerding and Mehlhose, 2020). In the present study, higher activation in the dlPFC meant that the PSAs had a greater impact on the participants and the ads were more effective. Therefore, we believe that the neural activity of dlPFC can predict the effectiveness of PSAs.

The products involved in v1 and v10 showed differences in the behavioral results of participants in group A and group B. This can be interpreted as viewers agreeing with the PSAs in their thoughts but not acting on them, consistent with the phenomenon mentioned by Kang et al. (2009). Combined with the interview results, we learned that this difference stems from the different content of the ads, which is consistent with the fact that ad content affects dlPFC activation (Wang et al., 2013). The different focus and presentation in the v1 and v10 resulted in different appeal of the ad content to participants, consistent with the findings of Shen (2015) on the effectiveness of sympathy appeals and fear appeals for anti-smoking ads. When people make decisions, they tend to value stimuli by using experiences and preferences as reference, showing activation of the dlPFC (Parnamets et al., 2020). From the interviews, we learned that the participants obtained some content of interest to them from v10, but did not obtain that from v1. Although the participants were unfamiliar with the fruits introduced by both v1 and v10, they had a reference in their value assessment because they got the information of interest from v10, as shown by the activation of the dlPFC, which was not the case with v1. It is noteworthy and interesting to note that most of the participants in group A bought the fruits presented in v1 and v10 out of curiosity and sympathy, because the anti-poverty project is a difficult and well-known project in China and most Chinese want to contribute to it. On advertising content alone, we believe that the activation of the dlPFC can be used as an indicator of the attractiveness of PSA content to enhance the advertising effect.

Although the results of this experiment are satisfactory, the shortcomings of this experiment still need to be addressed. The prediction method adopted in this experiment was within-sample prediction, and whether the experimental results can be applied to the overall population cannot be determined and still needs to be verified in future studies. Furthermore, although, we found that activation of the dlPFC can be used as an indicator of the effect of advertising, there are still some brain areas that we have not studied. McClure et al. (2004) suggested that vmPFC would assume a major role in decision-making and show greater activation when the decision is based on perception only; dlPFC would be more active when the information is more comprehensive. Wang et al. (2013) also suggested that difference in PSAs content can cause activation of the inferior frontal gyrus and precuneus. However, due to the limitation of the fNIRS observation range, we could not observe the neural activity of vmPFC, inferior frontal gyrus and precuneus. Whether advertising content affects emotion, whether emotion affects the decision-making process, and whether advertising content can be better improved through other brain regions need to be investigated further in future research.

## Research and Managerial Implications

Studies have shown that most consumer purchases are impulsive, that 70% of purchases occur within 60 s (Rook and Fisher, 1995), and that better planning of advertising content to influence consumer decisions is the key to improving advertising effectiveness (Fisher et al., 2010). The same effect is true for PSAs. The more appealing the content of a PSA, the higher the likelihood that it will influence people. Nowadays, there are many types of PSAs, anti-gambling ads (Shead et al., 2011), and healthy diet ads (Phua, 2014). The method used by these studies is still to evaluate the effectiveness of PSAs based on the people’s self-report. According to the results of this study, people’s neural activity can predict the effectiveness of PSAs more accurately than self-report, and the rational use of neuroimaging tools can better influence people’s behavior. The results of this study may provide a new approach for subsequent PSAs research to improve the effectiveness of advertising.

To change the bad social phenomenon and guide people’s behavior, the preparation of PSAs needs to speed lots of time and money. Ineffective PSAs cannot achieve their purpose, can only waste a lot of resources. The results of this study provide a method to predict the effectiveness of PSAs before they are released. Meanwhile, the appeal of content can be adjusted according to the neural activity of viewers, so as to improve their influence and avoid the waste of social resources.

## Conclusion

This study found that there was a positive correlation between the activation of dlPFC and the effectiveness of PSAs; The activation of dlPFC can also be used as an indicator of the attractiveness of the advertising content and help improve the effectiveness of PSAs. These findings imply that neuroimaging tools can be used not only in commercial advertising effectiveness and some PSA effectiveness studies, but also in PSA effectiveness studies in the remaining fields. Meanwhile, the findings of this study can serve as a primer for subsequent studies on the relationship between PSA effectiveness and neural activity. Before the PSAs are released, advertisers can predict the effect of the ads based on the audience’s dlPFC neural activation, determine the content of the ads that can better influence the audience, improve the effectiveness of the ads, and avoid wasting resources.

In this study, only dlPFC was investigated as an observed brain region, but there are many other brain regions associated with advertising effects, such as vmPFC and insula, according to previous studies. Limited by the measurement depth of fNIRS, we were unable to measure these brain regions, which may also have indicators related to advertising effects. Future research could start with two aspects: first, whether the brain regions that were not observed in this study are related to improving the effectiveness of PSAs; and second, the neural mechanisms by which emotions influence audience behavior and how emotions affect PSA effectiveness.

## Data Availability Statement

The datasets presented in this article are not readily available because it was ensured to the participants that their data is not available for third parties and it was guaranteed that participants can request the complete deletion of their datasets at any time. Requests to access the datasets should be directed to alixig@163.com.

## Ethics Statement

The studies involving human participants were reviewed and approved by College of Economics and Management Ethics Committee, Zhengzhou University of Light Industry. The patients/participants provided their written informed consent to participate in this study.

## Author Contributions

All authors listed have made a substantial, direct, and intellectual contribution to the work and approved it for publication.

## Funding

This study was supported by Science and Technology Project of Science and Technology Department of Henan Province (202102310305); Graduate Education Reform and Quality Improvement Project of Henan Province (HNYJS2020JD04); and General Project of Soft Science Research of Henan Province (192400410140).

## Conflict of Interest

The authors declare that the research was conducted in the absence of any commercial or financial relationships that could be construed as a potential conflict of interest.

## Publisher’s Note

All claims expressed in this article are solely those of the authors and do not necessarily represent those of their affiliated organizations, or those of the publisher, the editors and the reviewers. Any product that may be evaluated in this article, or claim that may be made by its manufacturer, is not guaranteed or endorsed by the publisher.

## Footnotes

^1^Experimental material source website: https://ggjzfp.cctv.com
